# Assessing the Impact of Simulated Color Vision Deficiency on Ophthalmologists’ Ability to Differentiate between Choroidal Melanoma and Choroidal Nevus

**DOI:** 10.3390/jcm13123626

**Published:** 2024-06-20

**Authors:** Yacoub A. Yousef, Fawzieh Alkhatib, Mutasem Elfalah, Saif Aldeen AlRyalat, Mona Mohammad, Omar AlHabahbeh, Reem AlJabari, Sandrine Zweifel, Ibrahim AlNawiaseh, Robert Rejdak, Mario Damiano Toro

**Affiliations:** 1 Department of Surgery (Ophthalmology), King Hussein Cancer Center, Amman 11941, Jordan; miss.alkhateeb@gmail.com (F.A.); dm.11804@khcc.jo (M.M.); omarhabahbeh.oh@gmail.com (O.A.); ra.11229@khcc.jo (R.A.); i-nawaiseh@khcc.jo (I.A.); 2 Division of Ophthalmology, Department of Special Surgery, School of Medicine, The University of Jordan, Amman 11942, Jordan; m.alrabie@ju.edu.jo (M.E.); s.alryalat@ju.edu.jo (S.A.A.); 3 Department of Ophthalmology, University of Zürich, 8057 Zürich, Switzerland; sandrine.zweifel@usz.ch; 4 Department of General and Pediatric Ophthalmology, Medical University of Lublin, 20-079 Lublin, Poland; robertrejdak@yahoo.com; 5 Eye Clinic, Public Health Department, University of Naples Federico II, 80131 Naples, Italy

**Keywords:** color blindness, choroid nevus, choroid melanoma, protanopia, deuteranopia

## Abstract

**Background:** Color vision deficiency (CVD) is an often-overlooked issue within the medical community, and its consequences remain insufficiently explored. We aim to evaluate how CVD affects diagnostic accuracy and distinguish between malignant choroidal melanoma and benign choroidal nevus among ophthalmologists. **Methods:** In this cross-sectional study, we engaged ophthalmologists through a web-based survey distributed via the professional ophthalmology society’s social media channels. The survey encompassed a series of three fundus images representing normal fundus, choroidal nevus, and choroidal melanoma. Each image underwent simulation for the three primary types of CVD—protanopia, deuteranopia, and tritanopia—alongside a non-simulated version. **Results:** The study included 41 participants, averaging 40 years of age (±9.2), comprising 28 (68%) men and 13 (32%) women. Significantly lower rates of identifying orange pigments were observed in simulated protanopia images compared to non-simulated ones (*p* = 0.038). In simulated deutranopia images, the recognition of melanotic lesions was notably reduced compared to non-simulated images (*p* = 0.048). No such limitation was observed for tritanopia. However, participants retained their ability to identify subretinal fluid and estimate tumor thickness in simulated and non-simulated images. Concerning simulated images of choroidal nevi, participants misdiagnosed nevi as choroidal melanoma in 37% of cases in simulated protanopia nevi images and 41% in simulated deutranopia nevi images. This resulted in unnecessary referrals of benign lesions as malignant, emphasizing the potential for mistaken diagnoses. Nevertheless, almost all simulated images of malignant melanoma were correctly referred for specialized oncological treatment. **Conclusions:** The simulated CVD conditions of protanopia and deuteranopia affected the accuracy of identifying the melanotic nature of the choroidal tumor and the presence of orange pigments. This limitation led to challenges in correctly diagnosing choroidal melanoma and choroidal nevus, resulting in extra referrals for nevus cases. However, participants were safe and could still determine the possible risk of eyes with choroidal melanoma, so most referred melanoma cases to specialized oncologists as needed.

## 1. Introduction

Color vision deficiency (CVD) is a prevalent visual impairment, affecting around 8% of men and 0.4% of women, with a higher incidence in males due to its X-linked recessive inheritance [1]. The most common form is red–green deficiency (deuteranopia), while total color blindness is rare [1,2]. In the medical field, global practices regarding CVD vary, with CVD not generally considered a barrier to entering medical training [1]. Medical school admission marks the formal application of health restrictions, although standards and accommodations for disabilities vary. Japan historically had strict entry policies, but concerted efforts led to a substantial reduction in exclusions [3]. In the United States, medical school standards outline essential abilities, including vague references to skills requiring vision, hearing, and touch [4]. The United Kingdom does not routinely screen medical school applicants for color deficiencies, while India does conduct routine screenings, therefore standardized actions for identified deficiencies are needed [5]. Notably, deficiencies in color vision may pose challenges in specific clinical tasks, but there is no evidence of an impact on overall clinical performance. Some publications advocate for the routine screening of medical students to enhance self-awareness, guide specialty choices, and mitigate potential errors [1]. CVD poses unique challenges in ophthalmology, where accurate color perception is pivotal for clinical examination and imaging interpretation. A few studies have highlighted the differences between healthcare professionals with CVD and those with normal color vision, indicating potential challenges in interpreting clinical images, leading to delayed referrals and treatment and increased morbidity and mortality rates for patients [1,6].

Uveal melanoma, constituting approximately 5% of all melanomas, is the most common primary intraocular malignancy [7]. The accurate diagnosis of choroidal tumors relies on clinical evaluation of various characteristics, including color, thickness, the presence of subretinal fluid, and other features. Color is a crucial diagnostic element, with melanomas exhibiting melanotic components of shades of brown or black and hemangiomas often appearing orange–red [8]. Imaging techniques, such as fundus photography, fluorescein angiography, fundus autofluorescence imaging, optical coherence tomography, and ultrasound imaging, play a crucial role in the diagnostic process [4,5,9,10]. The invasion of choroidal melanoma results in the accumulation of orange pigments (which needs to be clinically detected) and the leakage of serum beneath the retina, causing exudative subretinal fluid [11]. Choroidal nevi, classified based on size and height, are monitored due to concerns of malignant transformation and difficulties in differentiation from small choroidal melanomas. The risk of the malignant transformation of typical choroidal nevi is considered low, given their frequency and the rarity of choroidal melanomas [12].

As medical institutions globally grapple with the implications of CVD, a pressing question arises: How does CVD affect the diagnostic accuracy of ophthalmologists in distinguishing between benign and malignant melanotic choroidal tumors, and what are the consequences for patient care? This study examines the impact of CVD on ophthalmologists’ accuracy in diagnosing choroidal melanoma and nevus based on clinical features and their referral decisions. Previous research has indicated that participants showed significantly lower accuracy in diagnosing circumscribed choroidal hemangioma, nevus, melanoma, and metastasis when images were simulated to resemble protanopia and deuteranopia, but not when images were simulated to resemble tritanopia. With the simulated choroidal nevi images, participants incorrectly assigned protanopia and deuteranopia nevi images to malignant lesions [6]. The findings from this research will enhance our understanding of the challenges faced by ophthalmologists with CVD and may lead to improved clinical practice regarding diagnostic strategies.

## 2. Methods

### 2.1. Study Design and Participants

This study used a cross-sectional design and involved ophthalmologists working in Jordan. Ethical approval was obtained from the institutional review board at the King Hussein Cancer Center (22 KHCC 008), and participants consented before participating. The research followed the guidelines of the Helsinki Declaration. Data were collected through an online survey targeting general ophthalmologists, retinal specialists, and ocular oncologists practicing in Jordan.

### 2.2. CVD Simulation and Assessment

Participants were enrolled through an online form. Before the study, the rater confirmed that participants did not have CVD using an online Ishihara Test. Participants then completed a questionnaire about demographic variables (age, gender, occupation), specialty, and professional practice years. Afterward, three fundus images were shown to participants: normal fundus, choroidal nevus, and choroidal melanoma.

**Figure 1:**
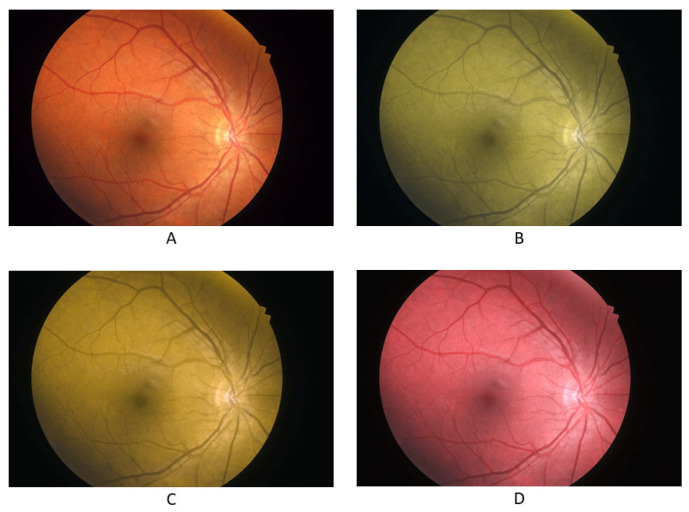
Fundus images for normal fundus (**A**) and images with simulated protanopia (**B**), deuteranopia (**C**), and tritanopia (**D**).

**Figure 2:**
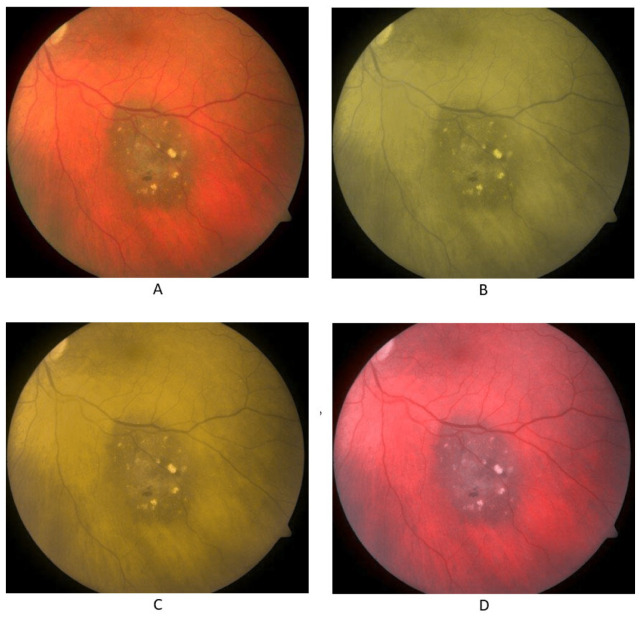
Fundus images for choroidal nevus (**A**) and images with simulated protanopia (**B**), deuteranopia (**C**), and tritanopia (**D**).

**Figure 3:**
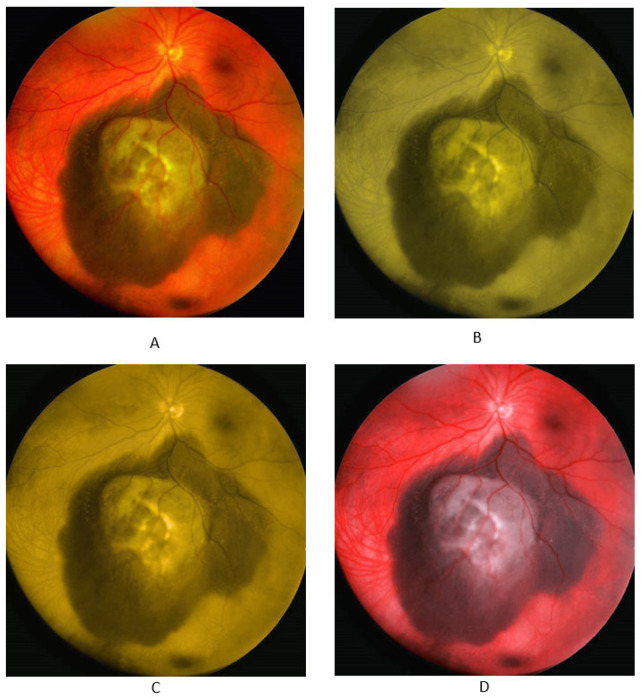
Fundus images for malignant melanoma (**A**) and images with simulated protanopia (**B**), deuteranopia (**C**), and tritanopia (**D**).

In addition to real photos of normal fundus, choroid nevus, and choroid melanoma, the participants were shown simulated color vision deficiency photos for normal fundus, choroid nevus, and choroid melanoma, with the photos simulating the three types of CVD: protanopia, deuteranopia, and tritanopia. These images were taken from the King Hussein Cancer Center database. Figure 1, Figure 2 and Figure 3 display the fundus images for normal fundus, choroid nevus, and choroid melanoma, alongside their simulated color deficiency variants.

In total, twelve fundus images were included and randomly distributed into four sets, with each comprising simulated protanopia, simulated deuteranopia, simulated tritanopia, and non-simulated images. Participants answered multiple-choice questions for each image, covering aspects such as identifying lesions, orange pigments, and subretinal fluid, estimating lesion thickness, determining whether the lesion was melanotic or amelanotic, providing a diagnosis, and indicating if referral to an ocular oncologist was needed. Participants who could not accurately diagnose the normal fundus photo in a non-simulated image were excluded from the study.

Using the Vischeck color blindness simulator in Fiji software, we accurately transformed fundus images into simulations that match the perception of protanope, deuteranope, and tritanope ophthalmologists [13,14].

### 2.3. Statistical Analysis

For our analysis, we employed SPSS version 26.0 (Chicago, IL, USA). We described continuous variables using mean (standard deviation) and nominal variables using count (frequency). We utilized Fischer exact tests to assess differences between specialties in the number of correctly diagnosed images. All underlying assumptions were met, and we considered a *p*-value of 0.05 as the threshold for statistical significance.

## 3. Results 

The study enrolled 41 participants with an average age of 40 years (SD = 9.2). Among them, 28 participants (68%) were male, while 13 participants (32%) were female. The majority of the participants (68%) were general ophthalmologists (n = 28), and the remaining 13 participants (32%) specialized in retinal diseases or ocular oncology. On average, the participants had 11 years of professional experience (±7.6), ranging from 1 to 35 years.

### 3.1. Melanoma Images

In non-simulated images, all participants (100%) correctly identified the presence of choroidal lesion. The lesion was correctly described as melanotic by 37 (90%) participants, orange pigments were recognized correctly by 31 (76%) participants, subretinal fluid was correctly recognized by 35 (85%) participants, and the thickness was correctly estimated to be more than 2 mm by 37 (90%) participants. The majority (38 participants; 93%) correctly gave the diagnosis of choroidal melanoma, and all (100%) of them decided this patient needed a referral to a specialized cancer care center for further evaluation and management. 

In simulated protanopia images, all participants (100%) correctly identified the presence of a choroidal lesion. The lesion was correctly described as melanotic by 32 (78%) participants, orange pigments were recognized correctly by 21 (51%) participants, subretinal fluid was correctly recognized by 36 (88%) participants, and the thickness was correctly estimated to be more than 2 mm by 38 (93%) participants. Only 30 (73%) participants correctly diagnosed choroidal melanoma. However, 39 (95%) participants decided this patient needed a referral to a specialized cancer care center for further evaluation and management. 

In simulated deuteranopia images, all participants (100%) correctly identified the presence of a choroidal lesion. The lesion was correctly described as melanotic by 29 (71%) participants, orange pigments were recognized correctly by 24 (58%) participants, subretinal fluid was correctly recognized by 33 (80.5%) participants, and the thickness was correctly estimated to be more than 2 mm by 36 (88%) participants. Only 30 (73%) participants correctly diagnosed choroidal melanoma. However, 40 (98%) participants decided this patient needed a referral to a specialized cancer care center for further evaluation and management. 

In simulated tritanopia images, all participants (100%) correctly identified the presence of a choroidal lesion. The lesion was correctly described as melanotic by 34 (83%) participants, orange pigments were recognized correctly by 13 (32%) participants, subretinal fluid was correctly recognized by 34 (83%) participants, and the thickness was correctly estimated to be more than 2 mm by 37 (90%) participants. Overall, 36 (88%) participants correctly gave the diagnosis of choroidal melanoma, and all (100%) participants decided this patient needed a referral to a specialized cancer care center for further evaluation and management. 

Overall, participants showed significantly lower recognition rates for orange pigments in simulated protanopia images than in non-simulated images (*p* = 0.038). Similarly, in simulated deuteranopia images, the identification of melanotic lesions was significantly lower than in non-simulated images (*p* = 0.048). Notably, most participants correctly diagnosed the lesion as choroidal melanoma in non-simulated images (n = 38; 93%). The accuracy rates for the correct diagnosis of choroid melanoma were low for simulated protanopia and deuteranopia images (73% for each condition); however, more than 95% of them realized the possible malignant nature of this lesion, which mandates referral to specialized centers for cancer care (Table 1). This indicates low diagnostic accuracy but high sensitivity to the possibly malignant nature of the lesion. 

### 3.2. Nevus Images

In non-simulated images, all participants (100%) correctly identified the presence of a choroidal lesion. The lesion was correctly described as melanotic by 39 (95%) participants, the absence of orange pigments was correctly recognized by 25 (61%) participants, the absence of subretinal fluid was correctly recognized by 31 (76%) participants, and the thickness was correctly estimated to be less than 2 mm by 32 (78%) participants. The majority (36 participants; 88%) correctly diagnosed choroidal nevus and 39 (95%) of them decided this patient should not be referred to a specialized cancer care center for further evaluation and management. 

In simulated protanopia images, all participants (100%) correctly identified the presence of a choroidal lesion. The lesion was correctly described as melanotic by 33 (80%) participants, the absence of orange pigments was correctly recognized by 34 (83%) participants, the absence of subretinal fluid was correctly recognized by 37 (90%) participants, and the thickness was correctly estimated to be less than 2 mm by 37 (90%) participants. Only 26 (63%) participants correctly gave the diagnosis of choroidal nevus, and 32 (78%) of them correctly decided that this patient does not need to be referred to a specialized cancer care center for further evaluation and management. 

In simulated deuteranopia images, all participants (100%) correctly identified the presence of a choroidal lesion. The lesion was correctly described as melanotic by 32 (78%) participants, the absence of orange pigments was correctly recognized by 23 (56%) participants, the absence of subretinal fluid was correctly recognized by 33 (80%) participants, and the thickness was correctly estimated to be less than 2 mm by 33 (80.5%) participants. Only 24 (59%) participants correctly gave the diagnosis of choroidal nevus and 30 (73%) of them correctly decided that this patient does not need to be referred to a specialized cancer care center for further evaluation and management.

In simulated tritanopia images, all participants (100%) correctly identified the presence of a choroidal lesion. The lesion was correctly described as melanotic by 38 (93%) participants, the absence of orange pigments was correctly recognized by 24 (59%) participants, the absence of subretinal fluid was correctly recognized by 32 (78%) participants, and the thickness was correctly estimated to be less than 2 mm by 30 (73%) participants. Only 34 (83%) participants correctly gave the diagnosis of choroidal nevus, and 38 (93%) of them correctly decided that this patient does not need to be referred to a specialized cancer care center for further evaluation and management.

Overall, the participants’ ability to recognize the absence of orange pigments for choroidal nevus in simulated protanopia images was significantly lower than in non-simulated images (*p* = 0.047). Similarly, in simulated deuteranopia images, the identification of the melanotic status of the lesion was notably reduced compared to non-simulated images (*p* = 0.048). Most participants accurately diagnosed the lesion as choroidal nevi in non-simulated images (n = 36; 88%). However, compared to non-simulated images, there were significantly lower accuracy rates for simulated protanopia (n = 26, *p* = 0.019) and deuteranopia (n = 24, *p* = 0.005). Regarding referral decisions, participants correctly recognized that the nevus lesion does not require referral in 95% (n = 39) of non-simulated images. However, participants mistakenly decided that nevi required referral to an ocular oncologist in simulated protanopia and deuteranopia, with nine (22%, *p* = 0.007) and eleven (27%, *p* = 0.003) participants, respectively, making this mistake (Table 2).

The accuracy rates for the correct diagnosis of choroid nevus were low for simulated protanopia (63%) and deuteranopia images (59%), and around 25% of these cases were mistakenly considered as malignant lesions and referred to specialized centers for cancer care (Table 2). This indicates low diagnostic accuracy and low sensitivity for the diagnosis of choroid nevus in simulated protanopia and deuteranopia images.

## 4. Discussion

This study explored the influence of CVD on ophthalmologists’ diagnostic accuracy and referral decisions for choroidal melanoma and choroidal nevi based on clinical features. The findings provide valuable insights into the challenges ophthalmologists with CVD may face in their clinical practice.

CVD is a significant concern among ophthalmologists, as accurate color perception is essential for detecting and diagnosing various ocular abnormalities. Our study revealed lower diagnostic accuracy rates for participants when presented with simulated protanopia and deuteranopia images of choroidal melanoma and nevus. These findings are consistent with previous research that highlighted a decrease in the diagnostic accuracy of circumscribed choroidal hemangioma, nevus, melanoma, and metastasis when images were simulated to mimic protanopia and deuteranopia as well as when determining whether choroidal nevi images contained a malignant lesion [6]. A noteworthy finding from our study was the tendency of participants to mistakenly refer choroidal nevus cases to ocular oncologists when presented with simulated protanopia and deuteranopia images. This misinterpretation could lead to unnecessary referrals, potentially overburdening healthcare resources and causing unnecessary patient stress. However, it is encouraging to note that participants demonstrated reasonable estimation skills for identifying subretinal fluid and estimating thickness in simulated and non-simulated images of choroidal melanoma and nevus. Additionally, accurate recognition and referral decisions were evident when participants viewed simulated tritanopia images, indicating a better diagnosis ability in people with tritanopia. 

The implications of CVD on ophthalmologists’ clinical practice extend beyond our study findings. A previously published study demonstrated lower staging accuracy for diabetic retinopathy among graders with CVD, especially among those with protanopia [15]. Additionally, physicians with CVD have shown reduced confidence in identifying certain clinical signs through colored photographs compared to those with normal color vision [16]. Colorblind ophthalmologists may face difficulties in diagnosing fundus pathologies due to altered color perception, which could lead to misinterpretations of pigmented lesions and retinal hemorrhages [17]. A colorblind ophthalmologist also described difficulties in diagnosing fundus pathologies, as normally pigmented red lesions appeared to him as bluish [3]. The reliance on digital cameras and algorithms to recreate fundus color images may not accurately represent reality, reinforcing the importance of their being examined ophthalmoscopically. Accurate interpretations are critical for the appropriate management of choroidal tumors, and any inaccuracies could result in suboptimal patient outcomes.

Physicians with CVD may lack awareness of the severity of their condition, and some may not even realize they have any deficiency at all [3]. The accurate recognition of colors is crucial for specific professions and necessitates pre-employment screening. Despite colorblindness testing being a prerequisite for medical school admission in some UK and all Taiwanese medical schools, it is not mandatory in most global medical institutions [18]. Incorporating screening procedures would enable the assessment of colorblindness severity. Counseling could be provided and individuals guided toward career paths that do not heavily rely on precise color identification. While establishing a direct link between medical errors due to colorblindness and patient care is intricate given the intricate decision-making in medical practice, this is recommended as a safety measure to prevent potential harm to patients. Our findings indicate that participants with color blindness had limited accuracy in diagnosing choroidal tumors. However, they could still safely discern whether the lesion was benign or malignant, thus mitigating patient harm.

The importance of accurate color vision in diagnosis extends beyond ocular tumors to involve skin lesions. This was previously elaborated in a study about skin lesions, where clinical features, including color, are essential for a clinical diagnosis [19]. For nearly 35 years, the ABCD pneumonic has guided the identification of malignant pigmented skin lesions, incorporating criteria such as asymmetry (A), border irregularity (B), color variegation (C), diameter over 6 mm (D), and the addition of evolution (E) [20,21,22,23,24]. Despite ongoing efforts to enhance accuracy, the influence of CVD on lesion recognition remains underexplored. That study aimed to assess how CVD affects the ability to distinguish between benign and malignant skin lesions, a crucial factor for early intervention [20,21,22,23,24]. The cross-sectional study involved final-year medical students and recent graduates, totaling 152 participants. After an Ishihara test and a Medscape presentation on lesion classification, participants completed a questionnaire assessing their ability to distinguish 20 nevi and 20 melanomas under protanope, deuteranope, and tritanope simulations and without simulation [13,25]. The results revealed that deuteranopia, associated with green color deficiency, significantly reduced accuracy in distinguishing lesions, particularly leading to the misclassification of benign lesions as malignant (*p* < 0.001). The impact was most pronounced in deuteranope simulations, with a mean of 32.2 (95% CI 27.0 to 37.6) higher accuracy for malignant lesions than benign ones. Moreover, notably, gender differences did not significantly affect classification accuracy across all simulations. The study emphasizes the crucial role of primary care physicians in lesion identification, highlighting that as accuracy decreases with CVD, more lesions may be categorized as malignant, potentially leading to necessary interventions [17,26]. That study concluded that deuteranopia substantially reduces accuracy in distinguishing pigmented skin lesions, particularly leading to the misdiagnosis of benign lesions as malignant. That study underscores the importance of recognizing CVD in physicians, especially in primary care settings where early lesion identification is crucial, and the authors recommended future studies with experienced dermatologists addressing device-related variabilities for a more comprehensive understanding [27,28]. Similarly, our previous study showed that simulated protanopia and deuteranopia CVD affected the accuracy of the diagnosis of several fundus lesions, including circumscribed hemangioma, choroidal nevus, choroidal melanoma and metastasis, and even normal fundus images. However, participants could still determine if the lesion was benign or malignant, which might warrant referral to specialist care [6]. Here, in this study, we showed that the simulated CVD conditions of protanopia and deuteranopia affected accuracy when identifying the melanotic nature of the lesion and the orange pigments in choroidal tumors, which affected the ability to distinguish choroidal melanoma from choroidal nevi.

This study has several limitations that should be considered in future research. Firstly, the number of cases in the survey was limited to prevent participant fatigue, and we selected clear-cut diagnostic cases to avoid confusion among general ophthalmologists. Secondly, we used simulated images instead of real-life scenarios, which may differ from the everyday practice of ophthalmologists. Thirdly, in real-life situations, ophthalmologists, particularly ocular oncologists, do not rely solely on clinical features for diagnosis. Multiple diagnostic modalities, such as ocular echography, FFA, FAF, and OCT (which shows subretinal fluids), are very helpful and essential. These tools can reduce the difficulties that color vision deficiency (CVD) ophthalmologists might face before diagnosing choroidal tumors. The value of these supporting features in the diagnostic accuracy of ophthalmologists with CVD for both oncological as well as non-oncological diseases of the retina deserves further exploration in subsequent studies. 

It is essential to acknowledge that the participants were aware they would be evaluating photos depicting specific scenarios, such as a normal fundus or classic examples of choroidal nevus or choroidal melanoma. This awareness could have influenced their diagnostic accuracy, potentially inflating the results compared to real-life situations with a broader range of possible diagnoses. These real-life scenarios might include amelanotic choroidal nevi, borderline melanocytic choroidal tumors, choroidal osteomas, retinal astrocytomas, vitreoretinal lymphoma, and various fundus lesions that can mimic malignant or benign fundus neoplasms (e.g., subretinal hematoma, localized suprachoroidal hematoma, inflammatory chorioretinal granuloma, and fundus lesions of sclerochoroidal calcification). Future studies should explore a broader range of choroidal lesions in the differential diagnosis to provide more realistic insights. Additionally, retinal diseases other than tumors (such as AMD and retinal dystrophies) may present features that are challenging for CVD ophthalmologists, such as hard and soft drusen, and these also need to be studied.

Another limitation of our study is the use of simulation software to mimic color vision deficiency (CVD), instead of testing actual color-blind practitioners. Although the software provides an approximation, it lacks standardization and validation to confirm its accuracy in replicating the real-life experiences of CVD. Future research should try to involve color-blind practitioners directly to validate these simulations and ensure our findings accurately reflect their diagnostic challenges.

## 5. Conclusions

In conclusion, CVD may impact an ophthalmologist’s ability to differentiate between choroidal melanoma and choroidal nevus. Estimation skills for subretinal fluid and thickness remained reasonable in both simulated and non-simulated images. The simulated CVD conditions of protanopia and deuteranopia affected the accuracy when identifying the melanotic nature of the lesion and orange pigments. This limitation led to challenges in correctly diagnosing choroidal melanoma and choroidal nevus, resulting in extra referrals for nevus cases. However, participants were safe and the possible risk of eyes with choroidal melanoma could still be determined, so most melanoma cases were referred to specialized oncologists as needed. Further investigations are needed to assess the clinical significance of these findings in individuals with CVD.

## Figures and Tables

**Table 1:** Responses based on color blindness patterns for melanoma.

	Non-Simulated Images’ Diagnosis Score		Simulated Protanopia Images’ Diagnosis Score		Simulated Deuteranopia Images’ Diagnosis Score		Simulated Tritanopia Images’ Diagnosis Score	
Overall (41 Participants)	Correct (%)	Wrong (%)	Correct (%)	Wrong (%)	Correct (%)	Wrong (%)	Correct (%)	Wrong (%)
Is there a lesson? Yes	41 (100%)	0 (0.0%)	41 (100%)	0 (0.0%)	41 (100%)	0 (0.0%)	41 (100%)	0 (0.0%)
	*p* value		1.0000		1.0000		1.0000	
Melanotic vs. Amelanotic	37 (90%)	4 (10)	32 (78%)	9 (22%)	29 (71%)	12 (29%)	34 (82.9%)	7 (17.1%)
	*p* value		0.22		0.048		0.518	
Orange Pigments Yes	31 (76%)	10 (24%)	21 (51%)	20 (49%)	24 (58.5%)	17 (41.5%)	13 (31.7%)	28 (68.3%)
	*p* value		0.038		0.15		0.1763	
Subretinal Fluid Yes	35 (85.4%)	6 (14.6%)	36 (87.8%)	5 (12.2%)	33 (80.5%)	8 (19.5%)	34 (82.9%)	7 (17.1%)
	*p* value		1.0000		0.7701		1.0000	
Estimated Thickness >2 mm	37 (90.2%)	4 (9.8%)	38 (92.7%)	3 (7.3%)	36 (87.8%)	5 (12.2%)	37 (90.2%)	4 (9.8%)
	*p* value		1.0000		1.0000		1.0000	
Diagnosis for Melanoma Yes	38 (93%)	3 (7%)	30 (73%)	11 (27%)	30 (73%)	11 (27%)	36 (88%)	5(12%)
	*p* value		0.0372		0.0372		0.712	

Need for Referral * Yes	41 (100%)	0 (0%)	39 (95%)	2 (%)	40 (98%)	1 (2%)	41 (100%)	0 (0%)
	*p* value		0.4938		1.0000		1.0000	

* Need to refer if they think it is malignant.

**Table 2:** Responses based on color blindness pattern for nevus.

	Non-Simulated Images’ Diagnosis Score		Simulated Protanopia Images’ Diagnosis Score		Simulated Deuteranopia Images’ Diagnosis Score		Simulated Tritanopia Images’ Diagnosis Score	
Overall (41 Participants)	Correct (%)	Wrong (%)	Correct (%)	Wrong (%)	Correct (%)	Wrong (%)	Correct (%)	Wrong (%)
Is there a lesson? Yes	41 (100%)	0 (0.0%)	41 (100%)	0 (0.0%)	41 (100%)	0 (0.0%)	41 (100%)	0 (0.0%)
	*p* value		1.0000		1.0000		1.0000	
Melanotic vs. Amelanotic	39 (95%)	2 (5%)	33 (80%)	8 (20%)	32 (78%)	9 (22%)	38 (93%)	3 (7%)
	*p* value		0.08		0.048		1.00	
Orange Pigments No	25 (61%)	16 (39%)	34 (83%)	17 (17%)	23 (56%)	18 (44%)	24 (59%)	17 (61%)
	*p* value		0.047		0.822		0.821	
Subretinal fluid No	31 (76%)	10 (24%)	37 (90%)	4 (10%)	33 (80%)	8 (20%)	32 (78%)	9 (22%)
	*p* value		0.1405		0.7902		1.0000	
Estimated Thickness <2 mm	32 (78%)	9 (22%)	37 (90.2%)	4 (9.8%)	33 (80.5%)	8 (19.5%)	30 (73.2%)	11 (26.8%)
	*p* value		0.2258		1.0000		0.7976	
Diagnosis for Nevus Yes	36 (88%)	5 (12%)	26 (63%)	15 (37%)	24 (59%)	17 (41%)	34 (83%)	7 (17%)
	*p* value		0.019		0.005		0.75	
Referral for Nevus * No	39 (95%)	2 (5%)	32 (78%)	9 (22%)	30 (73%)	11 (27%)	38 (93%)	3 (7%)
	*p* value		0.007		0.003		1.0000	

* Need to refer if they think it is malignant.

## Data Availability

Data are available upon reasonable request to the corresponding authors.
